# A Case Report on Penile Reconstruction to Correct Secondary Penoscrotal Webbing

**DOI:** 10.7759/cureus.91818

**Published:** 2025-09-08

**Authors:** Raghavendra Chintha, Manimaran R, Koppolu Kanchana

**Affiliations:** 1 General Surgery, Sree Balaji Medical College and Hospital, Chennai, IND; 2 Plastic Surgery, Sree Balaji Medical College and Hospital, Chennai, IND

**Keywords:** circumcision, penoscrotal webbing, reconstructive surgery, vy scrotoplasty, webbing

## Abstract

Circumcision of the penis is a common practice in different parts of the world. Although very common, this procedure is associated with non-favorable outcomes if not carried out by trained professionals or clinicians. Penoscrotal webbing, if not congenital, is a noted complication, usually associated with circumcision. Severe penoscrotal webbing requires attention due to its functional impairments. Surgical management is a routine practice for the correction of penoscrotal webbing. This is a case of a 19-year-old male presenting with difficulty voiding urine and penoscrotal webbing. He was managed with VY scrotoplasty. The patient has symptomatic relief and was found to be recovering well. This case aims to highlight the challenges in diagnosis and the need for early intervention and multispecialty involvement for managing such cases.

## Introduction

Penoscrotal webbing, also termed webbed penis, is the abnormal fusion of the skin of the penis and scrotum. Circumcision is the predominant reason for penoscrotal webbing; however, it can have either congenital or acquired etiology [1]. Penoscrotal webbing is characterized by the shifting of the scrotum onto the penis, which obscures the penoscrotal angle. This is caused by the aberrant dartos bands [2]. Scrotoplasty is primarily recommended for correcting penoscrotal webbing in cases involving pain during erection or cosmetic concerns [3]. The management of penoscrotal webbing is carried out by scrotoplasty with methods such as rotational flaps, Z-plasty techniques, inverted Y, VY scrotoplasty, and complete exteriorization of the shaft; however, the management completely depends on the deformity [2,4]. This is a case of a 19-year-old male with the primary complaint of difficulty voiding and a surgical history of two circumcisions. He was successfully managed with a VY scrotoplasty. 

## Case presentation

**Figure 1:**
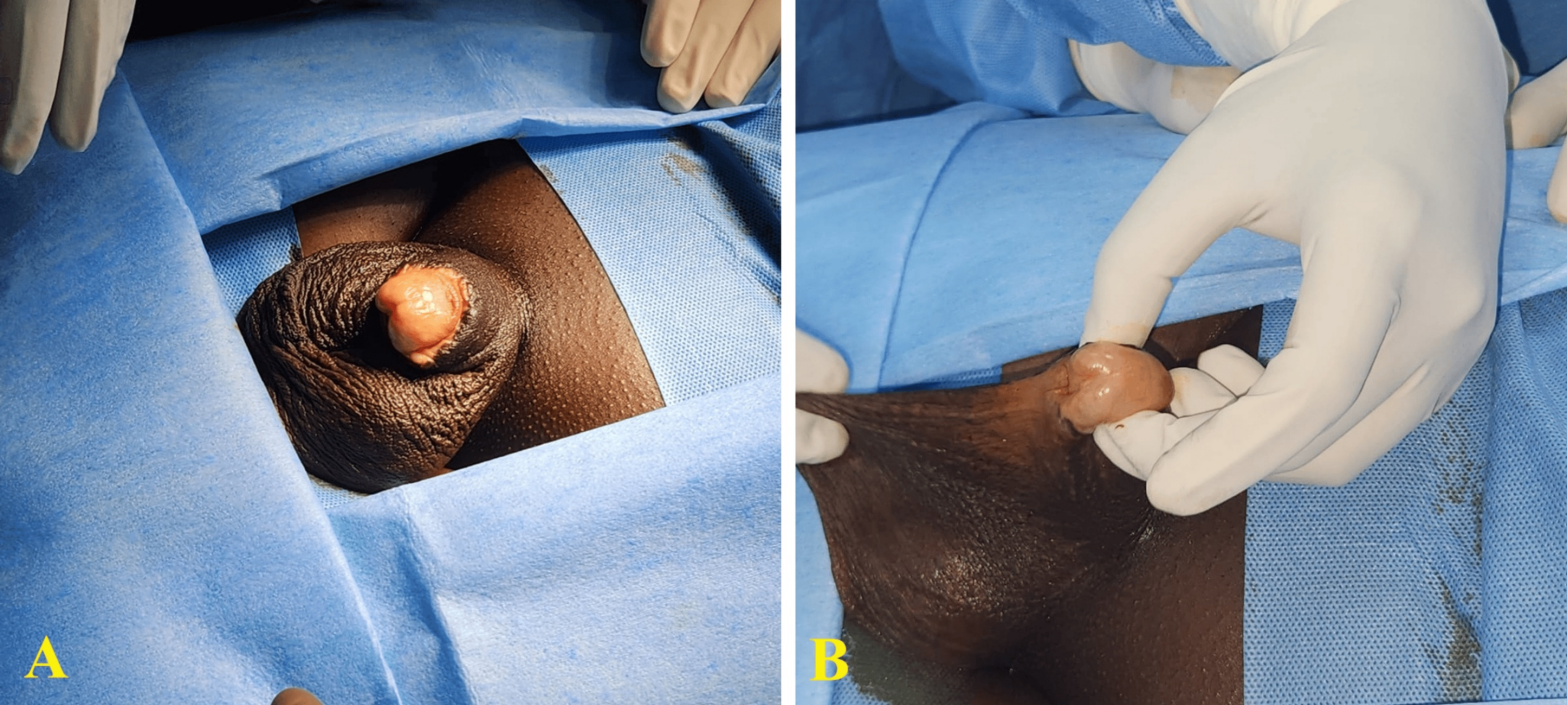
Physical presentation of the penis A: Dorsal view showing the short penile shaft, B: Ventral view showing the obscured penoscrotal angle

A 19-year-old male presented with the complaint of difficulty maintaining a stream of urine. The patient had a downward stream of urine leading to soiling of clothes because of the penoscrotal webbing caused by prior surgeries. There was no specific medical history except a surgical history of two circumcisions. The first circumcision was done at four years of age, followed by another one at 17 years of age. The second circumcision was carried out as the patient developed the same complaints. He was unable to retract the penile foreskin and had recurrent urinary tract infections for which he underwent re-circumcision. Both the procedures were carried out in a clinical setting. There was no meatal stenosis on clinical examination. No other associated risk factor was identified. Physical examination revealed penoscrotal webbing extending to the base of the penile shaft as a result of the earlier circumcisions (Figure 1).

**Figure 2:**
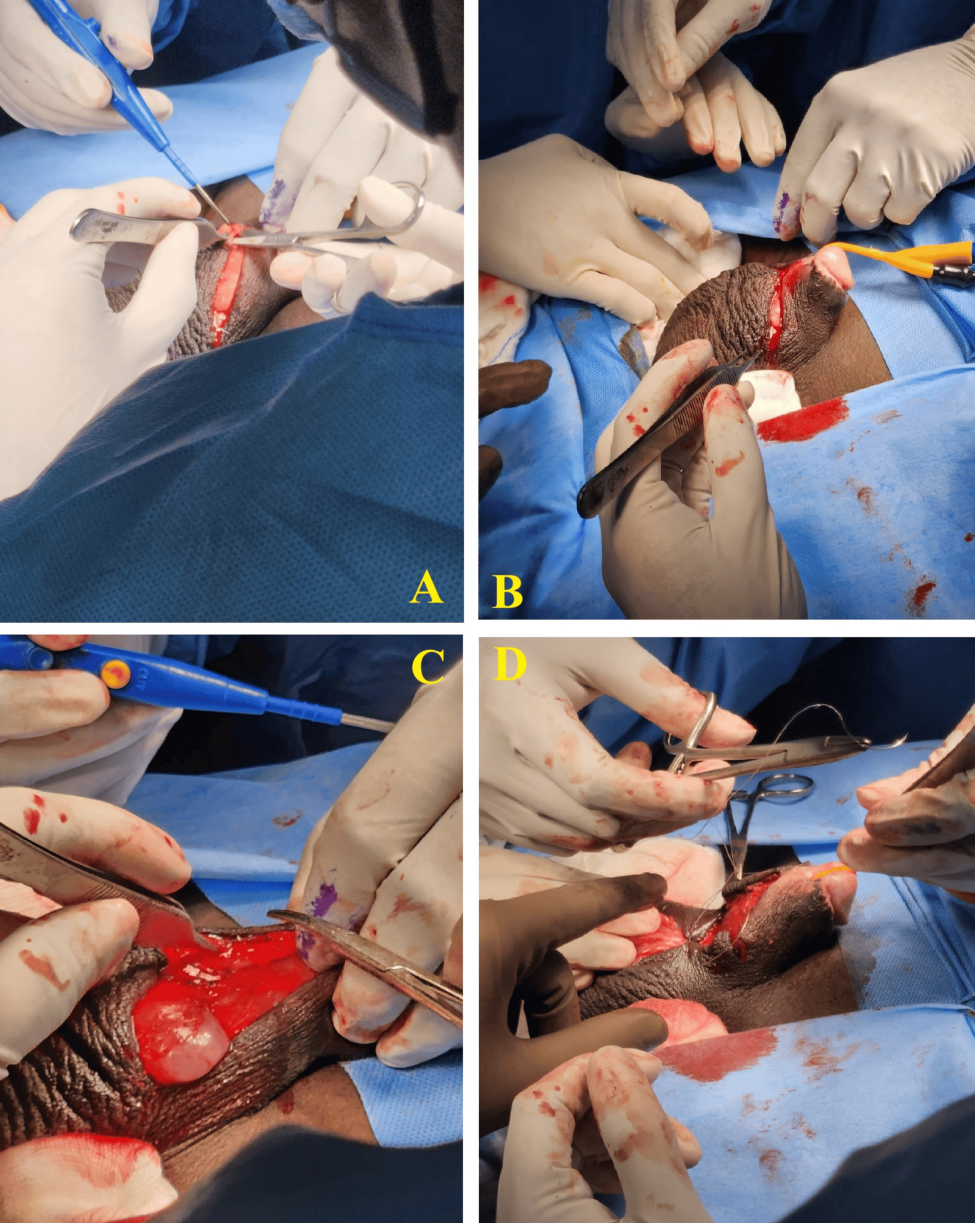
Intraoperative image showing the steps of the VY scrotoplasty A: V-incision over the penoscrotal web; B, C, and D: Deepening of incision and closure with adequate penile shaft length while maintaining penoscrotal recess

**Figure 3:**
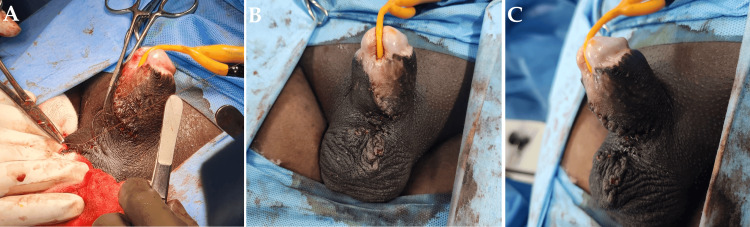
Intraoperative images post the correction procedure A and B: Ventral view, C: Lateral view of immediate post-procedure correction showing closure of Y incision and increased penile shaft length and penoscrotal angle

The patient was planned for surgical reconstruction to improve physiological function. The penile reconstruction was carried out by VY scrotoplasty with the primary aim of improving function and for cosmetic reasons. A V-shaped incision was made over the penoscrotal webbing with limbs facing towards the scrotum, and closure was done. This provides an adequate penile shaft length, and the penoscrotal recess is maintained. Hence, this technique was adopted. The patient was given regional anesthesia. A V-shaped incision on the ventral side of the penis was made, followed by the incision of the webbed skin in a longitudinal manner. This helped in obtaining a new angle between the penis and the scrotum. Incisions were made on both sides to release the penile-scrotal fusion in a downward direction. Any redundant or wrinkled skin was excised, and intermittent sutures were placed to close the area (Figures 2-3).

**Figure 4:**
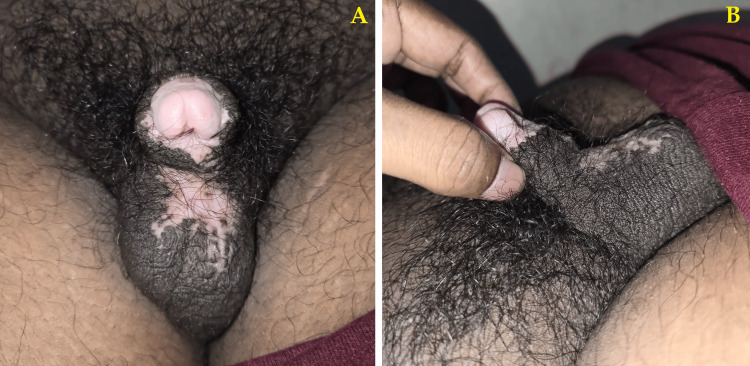
Images taken two months after the VY scrotoplasty The images from the second-month follow-up show increased penile shaft length and penoscrotal angle (A: Ventral view, B: Lateral view). The incision has healed well with primary intention, minimal scarring, and no noted webbing between the scrotal skin.

The patient was recovering well, with a noted improvement in penile shaft length and function as observed by no pain/difficulty in voiding urine. He was able to void urine without soiling his clothes, which was the primary complaint on admission. The patient was discharged on the second postoperative day. The first follow-up on the 15th postoperative day showed normal healing, with no signs of infection at the suture line. The patient was found to be recovering well with complete symptomatic resolution at the second-month follow-up with no new complaints (Figure 4).

## Discussion

Penoscrotal webbing can have either a congenital or acquired etiology. Though the causes for congenital penoscrotal webbing are unknown, acquired etiology is usually connected to circumcision, which is common in some regions due to religious and ritualistic practices [4,5]. Penoscrotal webbing can be noted in cases after circumcision when it is not performed correctly, especially by non-medical practitioners, leading to tethering of the scrotum to the penile shaft. Scrotal webbing can be caused by excessive circumcision when the penoscrotal junction is pulled distally along the shaft or excessive laxity of the scrotum or by a combination of both [4,6]. A similar finding was noted in this patient, who had a surgical history of two circumcisions, thereby developing severe penoscrotal webbing, causing difficulty in urination. Management of this condition depends on the severity of the clinical presentation, which is categorized into mild (grade 1, involving the proximal part of the penis), moderate (grade 2, involving the mid part of the penis), and severe webbing (grade 3, involving the distal part of the penis) [7]. 

Different surgical techniques are applied for the management, based on the grades of penoscrotal webbing. A transverse incision is made at the penoscrotal angle, and the defect is vertically closed using the Heineke-Mikulicz (HM) technique to treat minor penoscrotal webs [8]. The HM technique has been acknowledged for minor webbing as compared to VY or Z scrotoplasty and its modifications that are recommended for moderate to severe presentations [3,7,9]. As per recommendations from the Italian Society of Pediatric Surgery (SICP), together with the Italian Society of Pediatric Anesthesia (SARNePI) (2018), these surgeries can be performed as day surgeries under regional anesthesia with VY or multiple Z-plasty techniques [7,10,11]. A VY-scrotoplasty or Z-scrotoplasty can help correct functional complications during erection in young adults with a horizontal incision that is closed on the longitudinal axis [3,7,11,12]. A similar approach using VY-scrotoplasty under regional anesthesia was utilized in our patient, which was successful in terms of symptomatic relief and resolution of the primary complaint of difficulty urinating. This approach was also helpful in providing adequate penile shaft length with a maintained penoscrotal recess. 

There are research studies that conclude that if there are no symptomatic indications, surgical corrections can be prevented in children, which is contraindicated by other research that states a psychological or social pressure in these patients during their growing years [7]. Although each surgical approach to address has its own set of limitations, they must be risk-stratified before penile reconstruction.

## Conclusions

Penile reconstruction can significantly improve the quality of life for individuals with acquired or congenital penile defects, particularly in terms of improving both cosmetic and functional outcomes. Multidisciplinary care involving urologists, plastic surgeons, and mental health professionals is crucial for optimal outcomes. To obtain a consensus on the best surgical therapy in this field, more scientific evidence is needed. Current reconstructive techniques should also be refined through innovation.
